# Coupled CP Decomposition of Simultaneous MEG-EEG Signals for Differentiating Oscillators During Photic Driving

**DOI:** 10.3389/fnins.2020.00261

**Published:** 2020-04-09

**Authors:** Kristina Naskovska, Stephan Lau, Alexey A. Korobkov, Jens Haueisen, Martin Haardt

**Affiliations:** ^1^Communications Research Laboratory, Ilmenau University of Technology, Ilmenau, Germany; ^2^Institute of Biomedical Engineering and Informatics, Ilmenau University of Technology, Ilmenau, Germany; ^3^School of Computer Science, Australian Institute for Machine Learning, The University of Adelaide, Adelaide, SA, Australia; ^4^Institute for Radio-Electronics and Telecommunications, Department for Radio-Electronic and Telecommunication Systems, Kazan National Research Technical University named after A.N Tupolev-KAI, Kazan, Russia

**Keywords:** alpha band, electroencephalography, frequency entrainment, magnetoencephalography, simultaneous diagonalization, steady-state evoked response, tensor, theta band

## Abstract

Magnetoencephalography (MEG) and electroencephalography (EEG) are contemporary methods to investigate the function and organization of the brain. Simultaneously acquired MEG-EEG data are inherently multi-dimensional and exhibit coupling. This study uses a coupled tensor decomposition to extract the signal sources from MEG-EEG during intermittent photic stimulation (IPS). We employ the Coupled Semi-Algebraic framework for approximate CP decomposition via SImultaneous matrix diagonalization (C-SECSI). After comparing its performance with alternative methods using simulated benchmark data, we apply it to MEG-EEG recordings of 12 participants during IPS with fractions of the individual alpha frequency between 0.4 and 1.3. In the benchmark tests, C-SECSI is more accurate than SECSI and alternative methods, especially in ill-conditioned scenarios, e.g., involving collinear factors or noise sources with different variances. The component field-maps allow us to separate physiologically meaningful oscillations of visually evoked brain activity from background signals. The frequency signatures of the components identify either an entrainment to the respective stimulation frequency or its first harmonic, or an oscillation in the individual alpha band or theta band. In the group analysis of both, MEG and EEG data, we observe a reciprocal relationship between alpha and theta band oscillations. The coupled tensor decomposition using C-SECSI is a robust, powerful method for the extraction of physiologically meaningful sources from multidimensional biomedical data. Unsupervised signal source extraction is an essential solution for rendering advanced multi-modal signal acquisition technology accessible to clinical diagnostics, pre-surgical planning, and brain computer interface applications.

## 1. Introduction

Magnetoencephalography (MEG) and electroencephalography (EEG) are contemporary methods to investigate the function and organization of the brain. They, respectively, measure the magnetic flux and the electric potential at the head surface that are generated by simultaneous neuronal activity inside the brain. MEG-EEG data are inherently multi-dimensional, typically including the dimensions time, space (channels), modality (MEG, EEG), participant, and experimental condition. Simultaneously acquired MEG and EEG signals capture aspects of the same electric activity over time and can, therefore, exhibit coupling.

Tensor algebra has applications in signal processing, data analysis, blind source separation, and many more (Cichocki et al., 2015). The multidimensional signals are decomposed into rank one components according to the Canonical Polyadic (CP) decomposition (Kolda and Bader, 2009). Roemer and Haardt (2008, 2013) present a Semi-Algebraic framework for approximate CP decomposition via SImultaneous matrix diagonalization (SECSI) for the efficient and robust computation of the an approximate low-rank CP decomposition of noise corrupted data.

Many combined signal processing applications benefit from a coupled analysis based on the coupled CP decomposition (Becker et al., 2012; Acar et al., 2013, 2015; Rivet et al., 2015; Naskovska et al., 2017b; Sørensen and De Lathauwer, 2017a; Zou et al., 2017). The coupled CP decomposition jointly decomposes heterogeneous tensors that have at least one factor matrix in common. Detailed analysis of the computation of the coupled CP decomposition based on Alternating Least Squares (ALS) is presented in Farias et al. (2016) and Cohen et al. (2016). Farias et al. (2016) and Cohen et al. (2016) show that the computation of the coupled CP decomposition based on ALS is sensitive to different noise variances in the different tensors. An extension of the SECSI framework (Roemer and Haardt, 2008, 2013) to the coupled SECSI (C-SECSI) framework is proposed in Naskovska and Haardt (2016). The C-SECSI framework efficiently approximates the coupled CP decomposition of two noisy tensors that have at least one mode in common even in ill-posed scenarios, e.g., if the columns of a factor matrix are highly correlated. Moreover, the C-SECSI framework offers adjustable complexity-accuracy trade-offs and efficiently decomposes tensors with different noise variances without performance degradation.

Human scalp EEG signals contain characteristic frequencies, which can be partly related to cognitive processes. Their power and synchronization can vary with wakefulness, attention, age, disease, and in response to a sensory input (Klimesch, 1999). The dominant frequency peak in the spectrum is called the alpha rhythm and is traditionally expected between 7.5 and 12.5 Hz in adults (Klimesch, 1999). The second strongest is the theta rhythm, typically between 4 and 7.5 Hz. Whereas the frequencies of alpha and theta covary, their band powers are related to each other in a reciprocal way (Klimesch, 1999). Both, alpha and theta bands, are specifically related to cognitive and memory performance. Good performance at rest is associated with a tonic increase in alpha together with a decrease in theta. During event processing, a strong decrease in alpha together with an increase in theta indicates good performance (Klimesch, 1999). Desynchronization in the lower alpha band indicates attention, whereas a desynchronization in the upper alpha band is associated with semantic memory performance. Synchronization of theta reflects episodic memory and successful encoding of new information (Klimesch, 1999).

Intermittent photic stimulation (IPS) is a stimulation of the brain with repetitive light flashes that can drive oscillations in the brain. This is called the photic driving (PD) effect. The PD effect is widely used to assess effects of medication and for neurological diagnostics of, for example, epilepsy (Kalitzin et al., 2002). IPS can cause a frequency entrainment and a resonance effect. Frequency entrainment is characterized by the synchronization of brain rhythms to the photic stimulation (da Silva, 1991; Notbohm et al., 2016). The resonance effect is characterized by an increased amplitude of brain rhythms, such as alpha and theta, when the stimulation frequency coincides with their intrinsic frequencies. Photic driving has been reported to appear with stimulation frequencies up to 90 Hz (Herrmann, 2001). The strongest resonance appears around the individual alpha frequency (Mangan et al., 1993; Klimesch, 1999; Herrmann, 2001; Lazarev et al., 2001). A secondary resonance can be observed in the individual theta band of adults (Mangan et al., 1993; Klimesch, 1999) and more pronounced in children and adolescents (Klimesch, 1999; Lazarev et al., 2001).

Schwab et al. (2006) have performed the first investigation of frequency entrainment using simultaneously recorded MEG and EEG signals during IPS with frequency fractions of the individual alpha rhythm of each participant. In a subsequent comparable experiment, Salchow et al. (2016) have shown that a strong alpha resonance exists for a rod-type photo-receptor cell input at stimulation frequencies close to the individual alpha frequency peak and in the theta band. In this study, we consider the same experiment as in Salchow et al. (2016) using a time-frequency transformation (Wacker et al., 2011).

The objective of this study is to extract and differentiate signal sources from simultaneous MEG-EEG recordings during intermittent photic stimulation using a coupled tensor decomposition. We evaluate the capability of the proposed approach by comparing it to alternative methods using simulated benchmark data.

## 2. Methods

### 2.1. Tensor Algebra and Notation

Scalars are denoted either as capital or lower-case italic letters *A, a*. Vectors, matrices, and tensors are denoted as bold-faced lower-case ***a***, capital ***A***, and bold-faced calligraphic letters A, respectively. The superscripts ^T^, ^H^,^−1^, and ^+^ denote transposition, Hermitian transposition, matrix inversion, and Moore-Penrose pseudo matrix inversion, respectively. The operators ||.||_F_ and ||.||_H_ symbolize the Frobenius norm and the higher order norm, respectively. Moreover, an *n*-mode product between a tensor $A\in {\mathbb{C}}^{I_{1}\times I_{2}\ldots \times I_{N}}$ and a matrix $B\in {\mathbb{C}}^{J\times I_{n}}$ is denoted by $A\times_{n}B$, for *n* = 1, 2, …*N* (Kolda and Bader, 2009). A super-diagonal or or identity *N*-way tensor with dimensions *R* × *R*… × *R* is denoted as $I_{N,R}$. The *n*-mode unfolding of a tensor A is symbolized as ${\left[A\right]}_{\left(n\right)}$, and the *n*-th 3-mode slice is denoted with $A_{n}=A_{\left(.,.,n\right)}$. Fundamental tensor algebra concepts and definitions are provided in Kolda and Bader (2009), De Lathauwer et al. (2000), Cichocki et al. (2015), Comon et al. (2009), and Sidiropoulos et al. (2017).

The CP tensor decomposition is an extension of the Singular Value Decomposition (SVD) to multidimensional arrays (tensors). It decomposes a tensor into the minimum number of rank one components. For a low-rank tensor $X_{0}\in {\mathbb{C}}^{M_{1}\times M_{2}\times M_{3}}$ with rank *R* the CP decomposition is

$$\begin{array}{l} X_{0}=I_{3,R}\times_{1}F_{1}\times_{2}F_{2}\times_{3}F_{3}, \end{array}$$

where $F_{1}\in {\mathbb{C}}^{M_{1}\times R}$, $F_{2}\in {\mathbb{C}}^{M_{2}\times R}$, and $F_{3}\in {\mathbb{C}}^{M_{3}\times R}$ are the factor matrices (Kolda and Bader, 2009; Cichocki et al., 2015). In contrast to the SVD, the factor matrices resulting from the CP decomposition are not necessarily unitary, implying that the underling rank one components are not orthogonal to one another. Moreover, the CP decomposition is essentially unique under mild conditions.

Due to the uniqueness properties, the factor matrices of a CP decomposition can be identified up to a permutation and one complex scaling ambiguity per column. There are many different types of algorithms for the computation of the CP decomposition that can be categorized as follows: Alternating Least Squares (ALS), Gradient Descent (GD), Quasi-Newton and Nonlinear Least Squares (NLS) based algorithms as well as semi-algebraic approaches.

If two low-rank noiseless tensors $X_{0}^{\left(1\right)}\in {\mathbb{C}}^{M_{1}\times M_{2}^{\left(1\right)}\times M_{3}^{\left(1\right)}}$ and $X_{0}^{\left(2\right)}\in {\mathbb{C}}^{M_{1}\times M_{2}^{\left(2\right)}\times M_{3}^{\left(2\right)}}$ have the first mode in common, then they have a coupled CP decomposition defined as

(1) $$\begin{array}{l} X_{0}^{\left(1\right)}=I_{3,R}\times_{1}F_{1}\times_{2}F_{2}^{\left(1\right)}\times_{3}F_{3}^{\left(1\right)}\\ X_{0}^{\left(2\right)}=I_{3,R}\times_{1}F_{1}\times_{2}F_{2}^{\left(2\right)}\times_{3}F_{3}^{\left(2\right)}, \end{array}$$

where, $F_{1}\in {\mathbb{C}}^{M_{1}\times R}$, $F_{2}^{\left(i\right)}\in {\mathbb{C}}^{M_{2}^{\left(i\right)}\times R}$ and $F_{3}^{\left(i\right)}\in {\mathbb{C}}^{M_{3}^{\left(i\right)}\times R}$, *i* = 1, 2 are the factor matrices and *R* is the rank of the tensors. The coupled CP decomposition has even more relaxed uniqueness conditions than the CP decomposition. Some uniqueness properties for the coupled CP decomposition are available in Sørensen et al. (2015) and Zou et al. (2017).

The coupled CP decomposition jointly analyzes heterogeneous data sets or signals and identifies their shared underlying components. The facts that the heterogeneous signals can have different nature and dimensions and that the uniqueness properties are relaxed make the coupled CP decomposition a very practical tool for array (Sørensen et al., 2015, 2018; Sørensen and De Lathauwer, 2017a,b), audio (Zou et al., 2017), and biomedical signal progressing (Becker et al., 2012; Acar et al., 2013, 2015; Papalexakis et al., 2014; Rivet et al., 2015; Naskovska et al., 2017a,b; Van Eyndhoven et al., 2017).

Another extension of the SVD to multidimensional arrays is the higher order SVD (HOSVD) or Multi-linear SVD (De Lathauwer et al., 2000). The factor matrices resulting from the HOSVD are unitary matrices and they represent a unitary basis of the *n*-mode unfolding of the tensor, for *n* = 1, …*N* (for *N*-way tensors). Similar to the concept of truncated SVD, for a noise corrupted tensor a truncated HOSVD can be defined. The truncated HOSVD is a practical tool for noise suppression, dimension reduction, and signal subspace estimation (Haardt et al., 2008).

In case of noise corrupted tensors the truncated coupled HOSVD,

(2) $$\begin{array}{l} X^{\left(1\right)}=X_{0}^{\left(1\right)}+N^{\left(1\right)}\approx S^{\left[\text{s}\right],\left(1\right)}\times_{1}U_{1}^{\left[\text{s}\right]}\times_{2}U_{2}^{\left[\text{s}\right],\left(1\right)}\times_{3}U_{3}^{\left[\text{s}\right],\left(1\right)} \end{array}$$

(3) $$\begin{array}{l} X^{\left(2\right)}=X_{0}^{\left(2\right)}+N^{\left(2\right)}\approx S^{\left[\text{s}\right],\left(2\right)}\times_{1}U_{1}^{\left[\text{s}\right]}\times_{2}U_{2}^{\left[\text{s}\right],\left(2\right)}\times_{3}U_{3}^{\left[\text{s}\right],\left(2\right)}, \end{array}$$

can be calculated jointly, for the common mode using the economy-size SVD,

$$\begin{array}{l} \left[\begin{array}{cc} {\left[X^{\left(1\right)}\right]}_{\left(1\right)} & {\left[X^{\left(2\right)}\right]}_{\left(1\right)} \end{array}\right]=U_{1}^{\left[\text{s}\right]}\cdot \Sigma_{1}^{\left[\text{s}\right]}\cdot V_{1}^{\left[\text{s}\right]\text{H}}. \end{array}$$

In (2) and (3), $S^{\left[\text{s}\right],\left(1\right)}$ and $S^{\left[\text{s}\right],\left(2\right)}\in {\mathbb{C}}^{R\times R\times R}$ are the truncated core tensors and the loading matrices $U_{1}^{\left[\text{s}\right]}\in {\mathbb{C}}^{M_{1}\times R}$, $U_{2}^{\left[\text{s}\right],\left(i\right)}\in {\mathbb{C}}^{M_{2}^{\left(i\right)}\times R}$ and $U_{3}^{\left[\text{s}\right],\left(i\right)}\in {\mathbb{C}}^{M_{3}^{\left(i\right)}\times R}$ have unitary columns and span an estimate of the column space of the *n*-mode unfolding of $X^{\left(i\right)}$, for *n* = 1, 2, 3 and *i* = 1, 2, respectively.

### 2.2. Computation of the Coupled CP Decomposition

In order to compute the factors corresponding to the coupled CP decomposition, the existing algorithms for the computation of the CP decomposition have to be modified. For instance, the ALS algorithm can be extended to a coupled ALS (C-ALS) by taking into account that the common factor matrix can be jointly computed by means of concatenation. Another weighted version of the coupled ALS using normalization that can even support hybrid and noisy coupling is proposed in Farias et al. (2016). For the purpose of dimensionality reduction a compression based on the HOSVD can be used as a preprocessing step for ALS (Cohen et al., 2016). These ALS based algorithms are easy to implement, however, they have no convergence guarantee and can require many iterations.

Alternatively, the coupled CP decomposition can be computed based on a line search. The line search based algorithm CCP-MINF is available in Tensorlab 3.0 (Vervliet et al., 2016). Additionally, a NLS-based algorithm is available in Tensorlab 3.0 (Vervliet et al., 2016). The CCP-NLS algorithm is an iterative algorithm that computes updates of the factor matrices based on a Newton descent, which includes a linear approximation of the Hessian. A further approach is the semi-algebraic computation of the CP decomposition, which involves converting the CP model into a simultaneous matrix decomposition (SMD) followed by diagonalization in order to obtain the factor matrices (Sørensen et al., 2015; Naskovska and Haardt, 2016). The coupled SECSI algorithm (Naskovska and Haardt, 2016) is an efficient extension of SECSI (Roemer and Haardt, 2008, 2013; Roemer et al., 2012) that uses the tensor structure to construct not only one but the full set of possible SMDs jointly for both tensors.

**Figure 1:**
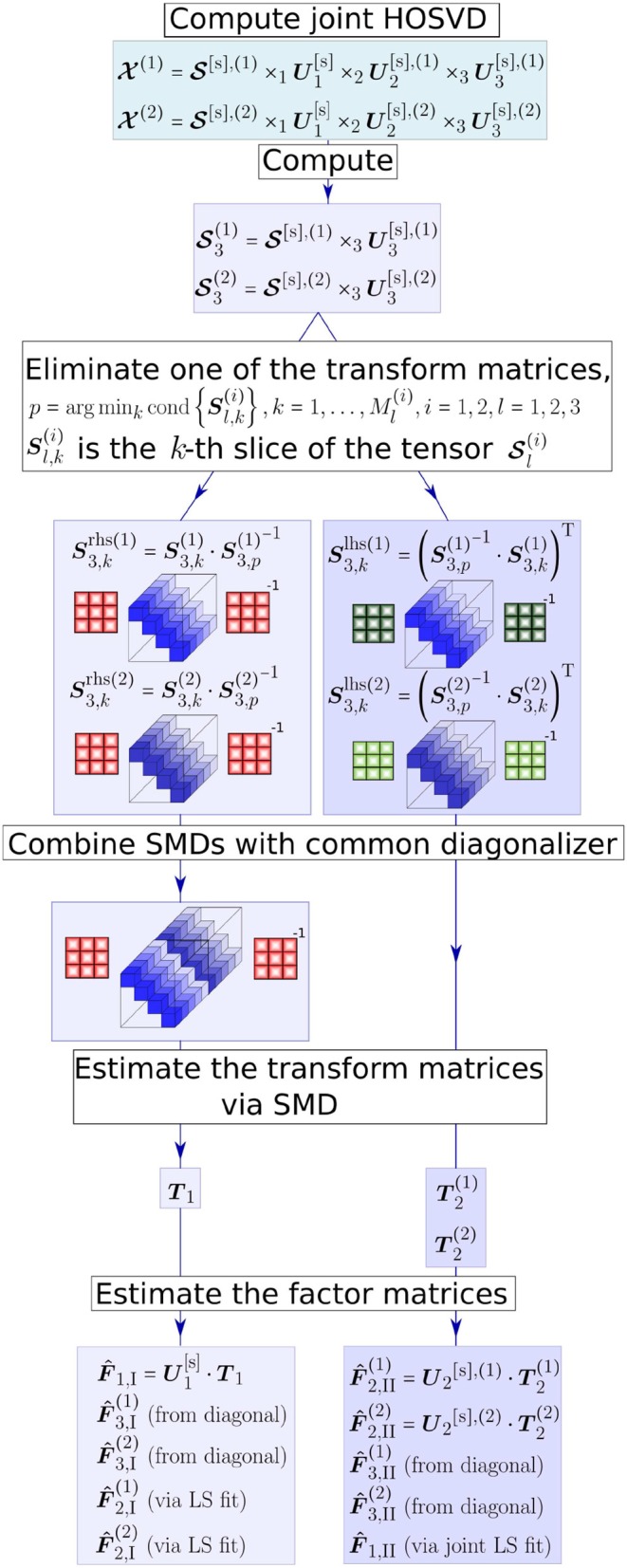
The C-SECSI framework for the computation of the coupled CP decomposition of two tensors $X^{\left(1\right)}$ and $X^{\left(2\right)}$ that have the first mode in common.

The C-SECSI approach provides an estimate of the factor matrices using the joint HOSVD followed by the whole set of possible SMDs. Eight initial estimates of the factor matrices I, …, VIII are obtained, if the two tensors have one factor matrix in common (Naskovska and Haardt, 2016). The computation of only two initial estimates of the factor matrices of two tensors $X^{\left(1\right)}$ and $X^{\left(2\right)}$ that have the first mode in common is visualized in Figure 1. These estimates are depicted by the two parallel branches in Figure 1, as well as an indication whether they are estimated from a transform matrix, from the diagonalized tensor, estimated via Least Squares (LS) or a joint LS fit. Note that these two estimates are obtained by diagonalizing the third mode of the core tensors resulting from the joint HOSVD, where ***T***_1_ and ***T***_2_ represent the transform matrices. Similarly, another two estimates of the factor matrices are obtained by diagonalizing the second mode of the core tensors. The diagonalization along the first mode (common mode) results in another four SMDs. However, since the common mode is in the diagonal elements, these SMDs cannot be combined. Therefore, they are solved separately and result in four additional initial estimates of the factor matrices.

From all these initial estimates of the factor matrices the one of major interest is the common factor matrix ${\hat{F}}_{1}$. The first four estimates of the common factor matrix (from ${\hat{F}}_{1,\text{I}}$ to ${\hat{F}}_{1,\text{IV}}$) are obtained either from the common transform matrices or via a joint LS fit. On the other hand, the last four estimates (from ${\hat{F}}_{1,\text{V}}$ to ${\hat{F}}_{1,\text{VIII}}$) are separately obtained from the diagonal elements of the diagonalized tensor. Therefore, the first four solutions are coupled and the last four are uncoupled. The final solution is then chosen for each of the tensors separately based on a heuristic that uses an accuracy-complexity trade-off (Roemer and Haardt, 2013; Naskovska and Haardt, 2016). Here, we use the reconstructed paired solutions (REC PS), with which the final solution is selected out of the eight estimates I − VIII based on the reconstruction error (Roemer and Haardt, 2013). The reconstruction error is calculated according to

$$\begin{array}{l} e_{\text{rec}}=\frac{||\hat{X}-X|{|}_{\text{H}}^{2}}{||X|{|}_{\text{H}}^{2}}, \end{array}$$

where $\hat{X}$ denotes the estimated tensor and X denotes the input tensor. Note that when X is a noisy observation as in **Figures 14**, **15**, i.e., $X=X_{0}+N$, we refer to this refer to this error as residual and denote it as RES.

To evaluate the reliability of the C-SECSI framework, we check whether the same (coupled) solution is chosen for both tensors (Naskovska et al., 2017a). Therefore, we define the reliability in percent

(4) $$\begin{array}{l} \text{REL}=\left(1-\frac{1}{2}\cdot \frac{||{\hat{F}}_{1}^{\left(2\right)}\cdot P-{\hat{F}}_{1}^{\left(1\right)}|{|}_{\text{F}}^{2}}{||{\hat{F}}_{1}^{\left(1\right)}|{|}_{\text{F}}^{2}}\right)\cdot 100\%, \end{array}$$

as a similarity measure of the final estimates of the common factor matrices. Here, ***P*** is a permutation matrix of size *R* × *R* that resolves the permutation ambiguity of the CP decomposition. Moreover, ${\hat{F}}_{1}^{\left(1\right)}$ and ${\hat{F}}_{1}^{\left(2\right)}$ are the final estimates of the common mode assigned to the tensors $X^{\left(1\right)}$ and $X^{\left(2\right)}$, respectively. This reliability measure has a maximum if the final estimates result from a coupled solution and the assumed rank is correctly approximated. Therefore, the reliability can be used to control the tensor rank of the coupled approximate CP decompositions. Note that for tensor rank one the reliability is always 100%. This is due to the fact that for rank one tensors the C-SECSI framework does not calculate any SMD. In this case, only one final estimate of the factor matrices is provided directly from the joint truncated HOSVD.

### 2.3. Measured MEG-EEG Signals

With ethics approval (Faculty of Medicine of the Friedrich-Schiller-University Jena, Germany), simultaneous MEG-EEG was performed on 12 healthy participants, aged between 21 and 42 years (median 26 years) during stimulation with flickering light (Salchow et al., 2016). The light stimulus was delivered through optical fibers from light emitting diodes outside the recording room. Light diffusers approximately 10 cm in front of the participants eyes provided a luminance of 0.0003 cd/m^2^. Throughout the exposure, the eyes of the participants were closed. The MEG provided 102 magnetometers and the EEG used 128 electrodes.

The purpose of the experiment was to investigate the behavior and interactions of oscillators in the healthy brain by systematically probing them with a controlled visual stimulation input. The frequency is a principle parameter of an oscillator. Therefore, a series of frequencies covering the alpha and theta bands was used to sample the brain's response pattern across the frequency dimension. The measured response is expected to contain multiple superimposed sources, some of which will be oscillatory. In a first step, the individual alpha rhythm was measured during 60 s of MEG at rest. The individual alpha frequencies *f*_α_ were then calculated by means of the averaged Discrete Fourier Transform (DFT) from the occipital MEG channels. The resulting alpha frequencies for Participants 1 to 12 are, in this order, 9.6, 10.7, 10.4, 10.8, 10.7, 10.8, 7.5, 10.8, 11.0, 10.7, 12.2, and 10.3 Hz.

IPS was then conducted at frequencies of *f*_s_ = [0.40 0.45 0.50 0.55 0.60 0.70 0.80 0.90 0.95 1.00 1.05 1.10 1.30 1.60 1.90 1.95 2.00 2.05 2.10 2.30] · *f*_α_. However, because there was no evidence of entrainment for stimulation frequencies larger than 1.30·*f*_α_ (Salchow et al., 2016), we used only the first 13 stimulation frequencies in this study, i.e., from 0.40·*f*_α_ until 1.30·*f*_α_.

Each stimulation frequency was performed in 30 stimulation trains, each consisting of 40 periods with an pulse/cycle duration of 0.5. Between each train there was a resting period of four seconds. From one frequency block to the next one, there was a resting period of 30 s. To avoid an ordering effect, the sequence of the stimulation frequencies was permuted and not known to the participant. Further details regarding the experiment including an analysis are provided in Salchow et al. (2016).

### 2.4. Signal Processing and Decomposition

The MEG and EEG signals were averaged for each stimulation frequency. A small number of non-functional EEG channels were excluded, which were typically at the inferior posterior edge of the EEG caps, where the variable head shape and/or thick hair layer results in the cap being too loose to fixate the electrodes and stabilize the electrolyte gel. These channels were identified based on their non-physiological signals, e.g., constant value, very strong noise or large artifacts. One technically faulty MEG sensor was excluded.

**Figure 2:**
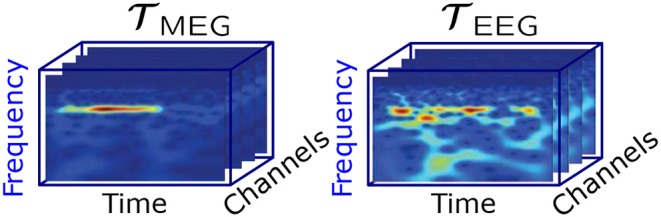
Visualization of the MEG and EEG tensor per participant and stimulation frequency.

A complex Morlet wavelet decomposition was used to obtain an estimate of the instantaneous frequencies across the stimulation time range of the MEG and EEG signals. Wavelet frequencies of 1,000/256 Hz, 1,000/255 Hz,…, 1,000/1 Hz were used during the decomposition. The wavelet coefficients between 3.77 Hz (1,000/265 Hz) and 15.15 Hz (1,000/66 Hz) were selected for the further analysis, thereby including the alpha and theta band. Figure 2 shows an example of the wavelet coefficients for each of the MEG and EEG channels arranged as slices in a three-way tensor.

As a result we have different complex tensors with dimensions frequency × time × channels for each stimulation frequency, measurement mode (MEG or EEG) and participant. The frequency and time dimensions correspond to the discretized values resulting directly from the wavelet transform. The frequency dimension contains 200 discrete frequency values from 3.77 Hz (1,000/265 Hz) to 15.15 Hz (1,000/66 Hz). The time dimension, however, varies from around 5,000 ms up to 20,000 ms depending on the corresponding stimulation frequency. Furthermore, the channel dimension represents the number of MEG and EEG channels, which varies across participants and conditions.

Next, the MEG and EEG signal tensors were jointly analyzed with the C-SECSI framework for each participant and stimulation frequency, respectively. The coupled CP decompositions were originally computed for different ranks. However, the reliability and the residual indicated that the tensor rank is overestimated for values equal to or larger than three (Naskovska et al., 2017a). Therefore, we assumed a tensor rank of two, $\hat{R}=2$. We assumed that the frequency mode is common for both, the MEG and the EEG signal tensor. Before the computation of the coupled CP decomposition, each of the tensors was normalized by calculating

$$\begin{array}{l} T_{\text{MEG}}^{\prime }=\frac{T_{\text{MEG}}}{||T_{\text{MEG}}|{|}_{\text{H}}}\text{ and }T_{\text{EEG}}^{\prime }=\frac{T_{\text{EEG}}}{||T_{\text{EEG}}|{|}_{\text{H}}}. \end{array}$$

This normalization of the tensors made the different amplitude scales and units (*fT* and μ*V*) of the MEG and EEG signals compatible.

### 2.5. Analysis of Components

The joint MEG-EEG signal decomposition based on the coupled CP decomposition resulted in three factor matrices for the MEG and three factor matrices for the EEG signal tensor [c.f. Equation (1)] for each participant and each stimulation frequency. Each factor matrix consisted of two columns corresponding to the two components due to the assumed rank $\hat{R}\;=\;2$.

The stimulation experiment provides us with a known reproducible visual response pattern that exhibits physiological integrity. However, the success of an individual experiment depends on the attention and compliance of the participant and varies across the series of repetitive stimulation. We aim to analyze the response of the brain to visual stimulation and, therefore, we need to determine the experiments in which that response was in fact successfully stimulated. For this purpose, the topographic distributions of the signals (field-maps) were labeled independently by three experienced professionals (SL, UG, DS). The measured MEG and EEG signals were converted to field-maps by calculating the root of the mean of the square (RMS) of the values in each channel. The components were displayed as field-maps by taking the channel factor matrix. We used three categories: (1) containing primarily only visual response patterns, (2) containing some visual response patterns and some other activity, and (3) containing no visual response patterns. For each participant, the labeler identified the participant-specific variation of the visual response pattern in position, orientation, symmetry and amplitude due to individual cortical folding, head shape and EEG cap/MEG placement. This could most easily be observed for stimulation close to the individual alpha frequency, where the response was typically strong and clear. Each labeler then labeled all data of that participant sequentially. The labeling sets were unified with a majority vote (if all three raters different, use Category 2). A majority was obtained in 96% of cases, including 88% perfect agreement and 8% with a deviation of one category step by one of the three labelers, e.g., if the dataset is between two categories. Note that this labeling takes into account the spatial characteristics of visual response signals and is more specific than a simple amplitude or power threshold in the alpha frequency band. Secondly, it accommodates the inclusion of sources with frequencies other than the alpha frequency, especially in the theta band.

For the group analysis of the brain oscillations during successful photic stimulation without confounding factors, we use only Category 1. For each component, the principal frequency, i.e., the maximum of the frequency signature, was determined as the obtained frequency of this component.

In order to separate the components reflecting recruitment to the stimulation frequency from other components, we differentiate two groups: The recruited group contains the components, whose obtained frequency is very close to the stimulation frequency or its second harmonic (Herrmann, 2001; Lazarev et al., 2001) with a maximum deviation of ±0.05*f*_s_ and ±0.05·2·*f*_s_, respectively. The non-recruited group consists of the remaining components. All frequencies are expressed in fractions of the individual alpha frequency of the participant to account for the inter-individual differences (Klimesch, 1999).

## 3. Results

### 3.1. Benchmark Performance With Simulations

In order to systematically compare the C-SECSI decomposition approach with other methods (Roemer and Haardt, 2008, 2013; Cichocki et al., 2015) on the algorithmic level, we use a set of simulations covering a broad range of tensor properties.

#### 3.1.1. Reliability

**Figure 3:**
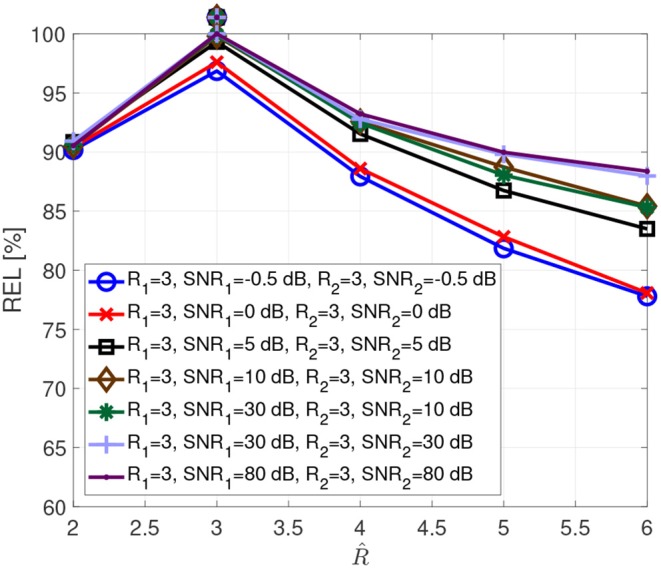
Reliability as a function of the assumed rank $\hat{R}$ for different SNRs.

Figure 3 visualizes the typical reliability as a function of the assumed rank $\hat{R}$. These curves result from Monte Carlo simulations with 1,000 realizations, for real valued tensors of dimensions 8 × 8 × 8, which spans open a tensor space with sufficient points per dimension to include complex multidimensional patterns. The entries of the factor matrices are drawn from a zero-mean Gaussian random process with variance one. Afterwards, the tensors are computed according to the coupled CP decomposition in Equation (1), allowing us to control the exact rank of the tensors. Additionally, a white Gaussian noise was added resulting in SNR_1_ (Signal to Noise Ratio) and SNR_2_.

$$\begin{array}{l} {\text{SNR}}_{1}=10\log_{10}\left(\frac{||X_{0}^{\left(1\right)}|{|}_{\text{H}}^{2}}{||N^{\left(1\right)}|{|}_{\text{H}}^{2}}\right)\;{\text{SNR}}_{2}=10\log_{10}\left(\frac{||X_{0}^{\left(2\right)}|{|}_{\text{H}}^{2}}{||N^{\left(2\right)}|{|}_{\text{H}}^{2}}\right) \end{array}$$

The SNR values in the simulations have been chosen to include realistic levels during physiological measurements, such as −0.5 dB for brain signals below noise level, 0 dB for brain signals at noise level and 5 dB for brain signals above noise but still impacted by it. The true tensor rank and the corresponding SNRs are indicated in the legend, while the assumed rank $\hat{R}$ is varied from two to six, given that several brain regions can be involved in performing a cognitive function. The true tensor rank for each curve is additionally indicated with a marker above the curves. It is clear that we have a reliability maximum when the assumed rank equals the correct tensor rank. Moreover, the SNR influences the reliability measure due to the dependency of the estimates on the SNR.

**Figure 4:**
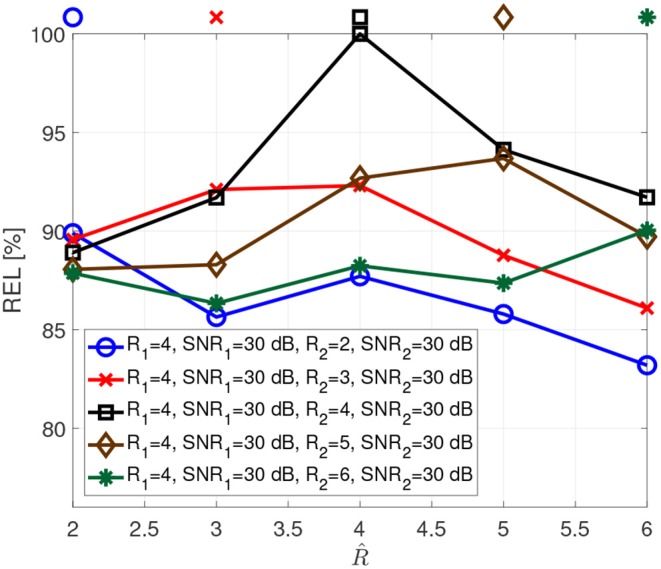
Reliability as a function of the assumed rank $\hat{R}$ for different ranks.

Figure 4 depicts the reliability when the two tensors have different numbers of components. For instance, for the blue curve the first tensor has rank 4, while the second tensor has rank 2. This implies that the tensors share only two components, and the first tensor has two additional components. In this case, the reliability has local maxima for both ranks.

#### 3.1.2. Accuracy

When performing a signal analysis using the CP decomposition, we are interested in the factor matrices, because their columns represent the signatures of the underlying components for the corresponding dimensions. Therefore, an important measure for the comparison of the algorithms is the total squared factor error (TSFE)

$$\begin{array}{l} \text{TSFE}=\frac{1}{N}\overset{N}{\underset{n=1}{\sum }}\underset{P\in M_{\text{PD}}\left(R\right)}{\min}\frac{||{\hat{F}}_{n}\cdot P-F_{n}|{|}_{\text{F}}^{2}}{||F_{n}|{|}_{\text{F}}^{2}}, \end{array}$$

where $M_{\text{PD}}\left(R\right)$ is a set of permutation matrices ***P*** of size *R* × *R*, *R* is the tensor rank, and *N* is the tensor dimensionality.

**Figure 5:**
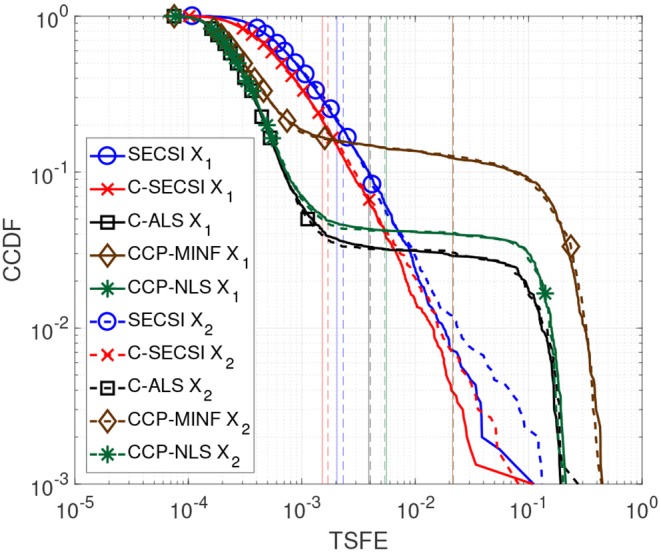
CCDF of the TSFE for real-valued tensors $X_{1}$ and $X_{2}$ with dimensions 40 × 4 × 10, tensor rank *R*_1_ = *R*_2_ = 3, factor matrices with mutually correlated columns designed as sine functions, and SNR_1_ = SNR_2_ = 25 dB.

In Figure 5 we compare the performance of C-SECSI (Naskovska and Haardt, 2016), SECSI (Roemer and Haardt, 2013), C-ALS, CCDP-NLS and CCDP-MINF (Vervliet et al., 2016) for two real-valued tensors of size 40 × 4 × 10, *R*_1_ = *R*_2_ = 3, with first mode in common. The three signatures of the first factor matrix represent the first 40 samples of sine functions, $\sin\left(2\pi tf_{1}+\frac{\pi }{3}\right)$, $\sin\left(2\pi tf_{2}\right)e^{t10\text{Hz}}$, and $\sin\left(2\pi tf_{3}\right)e^{-t3\text{Hz}}$ with *f*_1_ = 10 Hz, *f*_2_ = 20 Hz, and *f*_3_ = 30 Hz, which are in the physiological frequency range for brain signals. The second and the third factor matrices are drawn from a zero-mean Gaussian random process with variance one. Moreover, the third factor matrices have collinear columns with a correlation factor of 0.9. The Complementary Cumulative Distribution Function (CCDF) of the TSFE for a SNR equal to 25 dB is depicted in Figure 5. The vertical lines represent the mean value of the error for each curve. SECSI and C-SECSI do not have outliers even for such an ill-conditioned scenario in contrast to the other algorithms.

**Figure 6:**
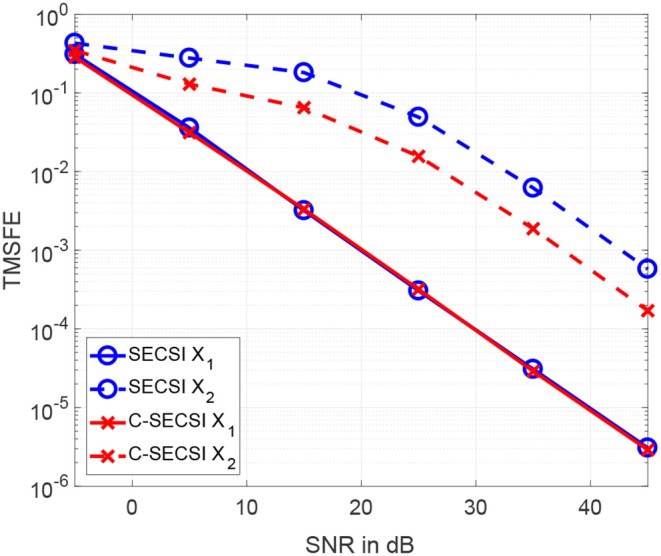
TMSFE as a function of the SNR for complex-valued tensors $X_{1}$ and $X_{2}$ with dimensions 4 × 8 × 7, tensor rank *R*_1_ = *R*_2_ = 3, where the second tensor has third factor matrix with mutually correlated columns.

The total mean squared factor errors (TMSFE) for different noise variances when using the uncoupled SECSI framework vs. the C-SECSI framework are presented in Figure 6. Both tensors $X_{1}$ and $X_{2}$ with common first mode have dimensions 40 × 4 × 10 and tensor ranks *R*_1_ = *R*_2_ = 3. The tensors are designed in the same manner as for Figure 5. However, only the third factor matrix of the second tensor $X_{2}$ has mutually correlated columns with a correlation coefficient of ρ = 0.98. This highly correlated factor matrix causes the tensor $X_{2}$ to be ill-conditioned. The Figure 6 shows that using the coupled algorithm improves the estimation accuracy of the ill-conditioned tensor without corrupting the well-conditioned tensor.

#### 3.1.3. Normalization

**Figure 7:**
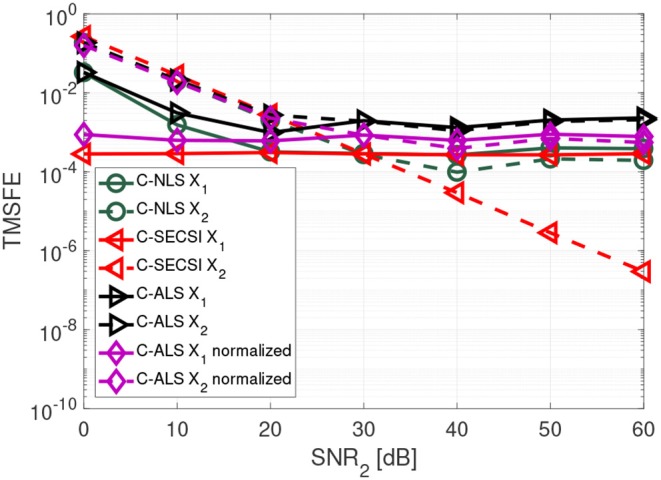
TMSFE as a function of the SNR_2_ for complex-valued tensors $X_{1}$ and $X_{2}$ with dimensions 3 × 8 × 7, tensor rank *R*_1_ = *R*_2_ = 3, and SNR_1_ = 30 dB.

In Figure 7 we show that the C-SECSI framework, unlike other algorithms, can jointly decompose coupled tensors even if they are affected by noise with different variance. The tensors $X_{1}$ and $X_{2}$ with common first mode have dimensions 3 × 8 × 7, and tensor ranks *R*_1_ = *R*_2_ = 3. The factor matrices contain complex values drawn from a zero mean circularly symmetric complex Gaussian random process with variance one. The first tensor has a constant SNR_1_ of 30 dB, while the SNR_2_ of the second tensor is varied from 0 to 60 dB. These results are averaged over 3,000 realizations. “C-ALS normalized” denotes the C-ALS algorithm with additional normalization with respect to the different noise variances. C-SECSI outperforms the “C-ALS normalized.”

Further details on the importance of normalization and compression with the HOSVD for ALS are available in Cohen et al. (2016). However, the results in Figure 7 show that normalization with respect to the noise variance is not required when computing the coupled CP decomposition using the C-SECSI framework.

### 3.2. Decomposition of Measured MEG-EEG Data

#### 3.2.1. Visual Response Rates

The labeling of visual response topographies in the RMS field-maps shows that 74% of the MEG measurements and 89% of the EEG measurements contain some visual response pattern (Categories 1 and 2). After the decomposition, there is some visual response pattern in at least one of the components in 73% of MEG measurements and 96% of EEG measurements. Clear visual response patterns (Category 1) are observed in 42% of the MEG data sets before the decomposition and 63% after the decomposition. Of the EEG data sets, 72% show clear visual response patterns before the decomposition and 85% after the decomposition. Within the components exhibiting a clear visual response pattern (Category 1), 32% of MEG components and 49% of EEG components belong to the recruited group, meaning that their dominant frequency matches the stimulation frequency or its first harmonic. The remaining data sets formed the non-recruited group, containing signal sources that had a dominant frequency that was different from the stimulation frequency.

#### 3.2.2. Characteristics of Estimated Components

**Figure 8:**
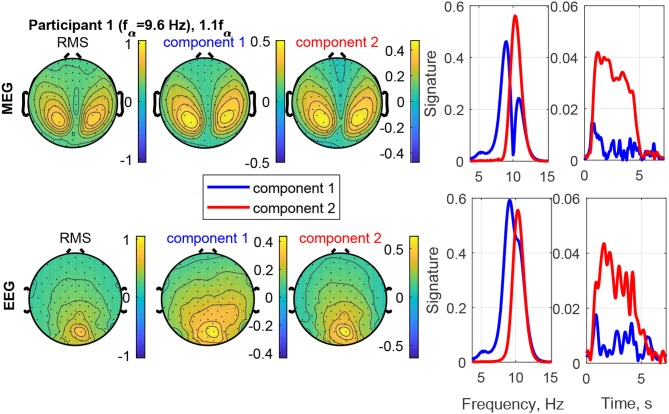
RMS, channel, frequency, and time signature for Participant 1 and stimulation frequency 1.1*f*_α_.

The estimated factor matrices from the coupled CP decomposition for Participant 1 and stimulation frequency 1.1*f*_α_ are shown in Figure 8. Additionally, the figure depicts the field-maps of the RMS of the measured signal for both, MEG and EEG (column 1). The RMS field-maps represent the power distribution of the measured signals before the decompositions. Columns 2 and 3 show the field-maps for the channel signatures for components 1 and 2, respectively. The frequency and time signatures are provided in columns 4 and 5. The stimulation with 1.1*f*_α_ produces the strongest response for Participant 1. Note that *f*_α_ is estimated from a resting state measurement and may, therefore, slightly differ during PD. In the RMS maps of MEG and EEG, we can see a clear occipital activation due to the PD (visual response Category 1). Both components are located in the occipital area as well and present two frequencies or narrow frequency distributions close to the individual alpha frequency of 9.6 Hz. Component 2 is common between the MEG and the EEG signals and matches closely the stimulation frequency (recruited). In the time domain (column 5) it is dominant and displays an onset, plateau, and offset phase. Component 1 is a visual response as well, but has a different obtained frequency (non-recruited) that is closer to the individual alpha frequency. In the time domain it is stronger in the onset phase of the stimulation train and diminishes after that.

**Figure 9:**
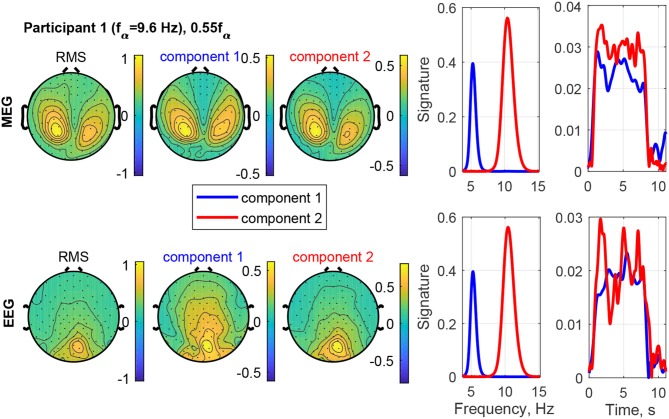
RMS, channel, frequency, and time signature for Participant 1 and stimulation frequency 0.55*f*_α_.

**Figure 10:**
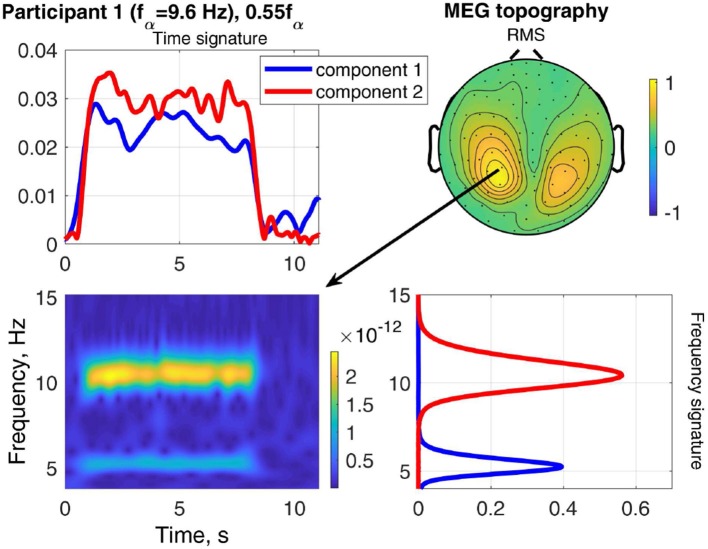
Relation between frequency distribution (bottom left) of an indicative MEG channel (top right) and the time (top left) and frequency (bottom right) signatures of the components of Participant 1 at stimulation frequency 0.55*f*_α_.

**Figure 11:**
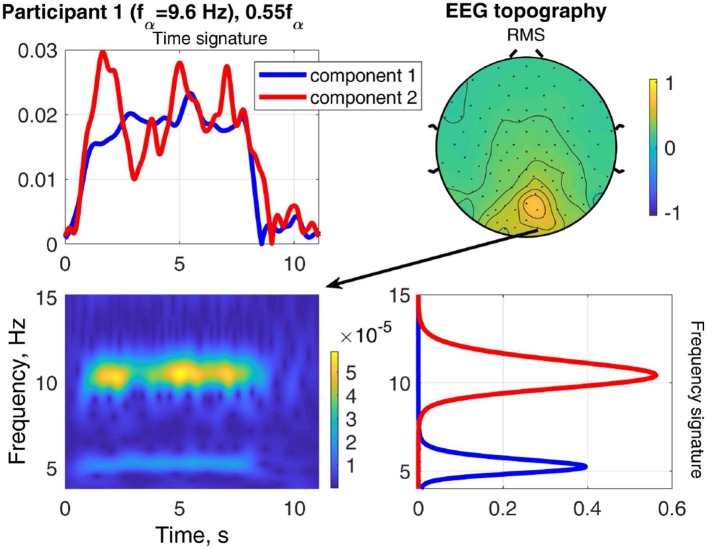
Relation between frequency distribution (bottom left) of an indicative EEG channel (top right) and the time (top left) and frequency (bottom right) signatures of the components of Participant 1 at stimulation frequency 0.55*f*_α_.

The result of stimulating the same participant with 0.55*f*_α_, in the theta band, is shown in Figure 9. This stimulation produced an increased response as well, which was not as strong as that of 1.1*f*_α_. The two components are both in the occipital region, show visual response patterns, and are both of comparable strength over the time course. Figure 10 shows the input frequency distribution of the MEG over time (bottom left) of an indicative channel (top right), the time signatures of the resulting components aligned above and the frequency signatures aligned on the right. Component 1 presents closely the stimulation frequency (recruited), which is visible in the frequency distribution during the stimulation train. Component 2 presents closely the harmonic 2·0.55*f*_α_ of the stimulation frequency (recruited). Figure 11 shows that both of these frequencies appear in the MEG and EEG signals. Component 1 of the EEG signal extends more centrally (Figure 9, row 2 column 2), which is consistent with the topography of theta band activity (Klimesch, 1999, p. 180).

**Figure 12:**
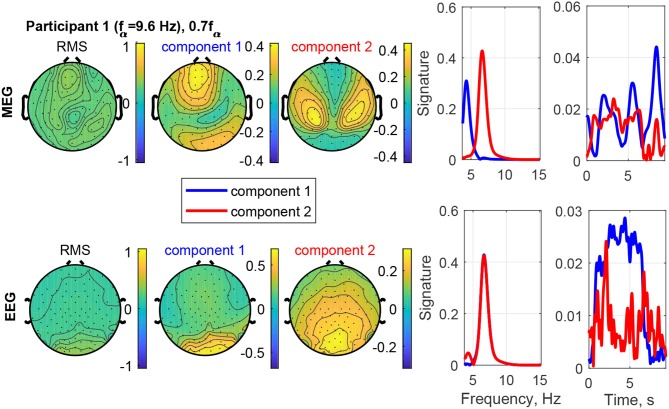
RMS, channel, frequency, and time signature for Participant 1 and stimulation frequency 0.7*f*_α_.

**Figure 13:**
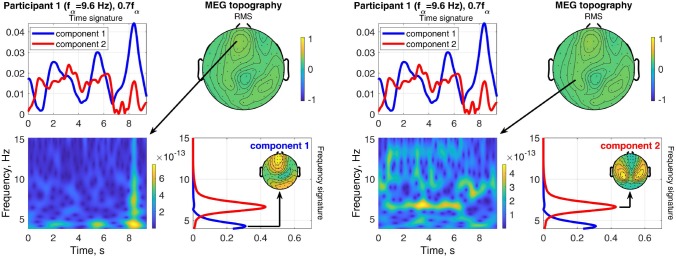
Relation between frequency distributions of one indicative MEG channel of component 1 (left sub-figure) as well as one indicative MEG channel of component 2 (right sub-figure) and the time and frequency signatures of Participant 1 at stimulation frequency 0.7*f*_α_.

Figure 12 shows an example of the separation of a visual response from a superimposed other source in Participant 1 at the stimulation frequency 0.7*f*_α_. The field-map of the MEG signals shows no clear visual response pattern (visual response Category 3). However, Component 2 reveals the visual response at the stimulation frequency (recruited). The other, more complex and temporally variable source is found in Component 1. Figure 13 shows the frequency distributions of the input MEG signal of a frontal channel primarily capturing the activity of Component 1 (left sub-figure) and an input MEG channel primarily capturing the activity in Component 2 (right sub-figure). The components reflect the primary frequency aspects, keeping in mind that the decomposition captures the primary aspects present across channels and time. The time signature of Component 2 matches the stimulation train, which further confirms that it is a stimulation response. The time signature of Component 2 is irregular and present after the stimulation train. This further confirms that it is not a direct stimulation response. The decomposition of the EEG signals differentiates two sources, which are both at the stimulation frequency (recruited). Component 1 is confined to the occipital region and dominates the time course, while Component 2 (red) includes a small fraction of activity in the theta band, extends more central and is more distributed.

The components of unsuccessful stimulation experiments, during which no consistent visual response could be elicited (data not shown), isolate the main patterns in the respective frequency distribution. These may be transient in time or only present at a short time point on the time axis, which is captured by the time signatures. The topographies of the signals and components are broader and not restricted to the occipital regions of the visual cortex. The signals may be generated by resting-state brain rhythms.

#### 3.2.3. Group Analysis of Obtained Frequencies

**Figure 14:**
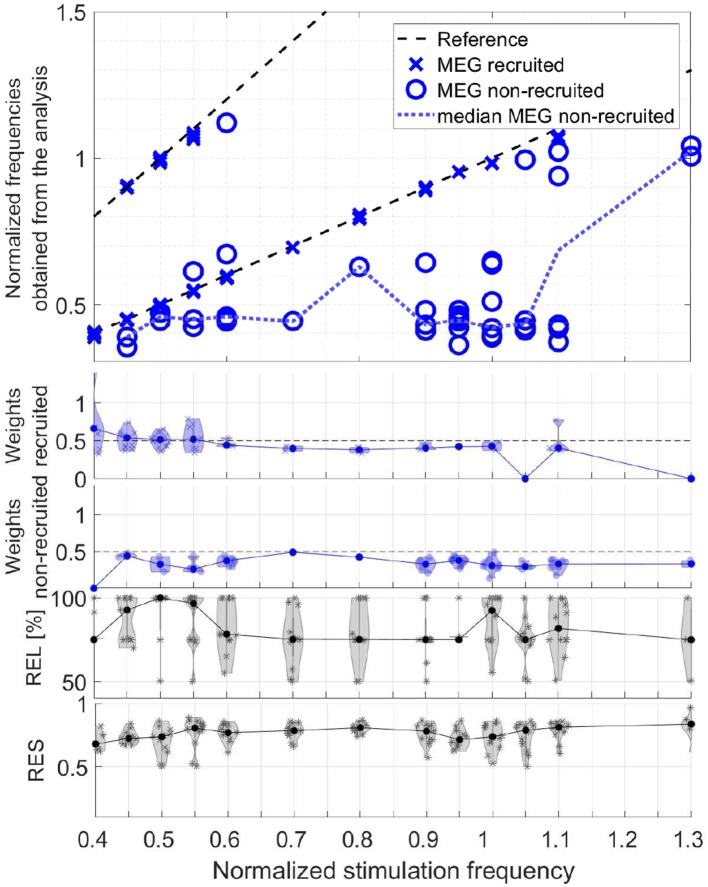
Scatter plot of the normalized frequencies obtained from the analysis for MEG data, violin plots of the weights of the recruited and non-recruited components, reliability, and residual as function of the normalized stimulation frequency.

**Figure 15:**
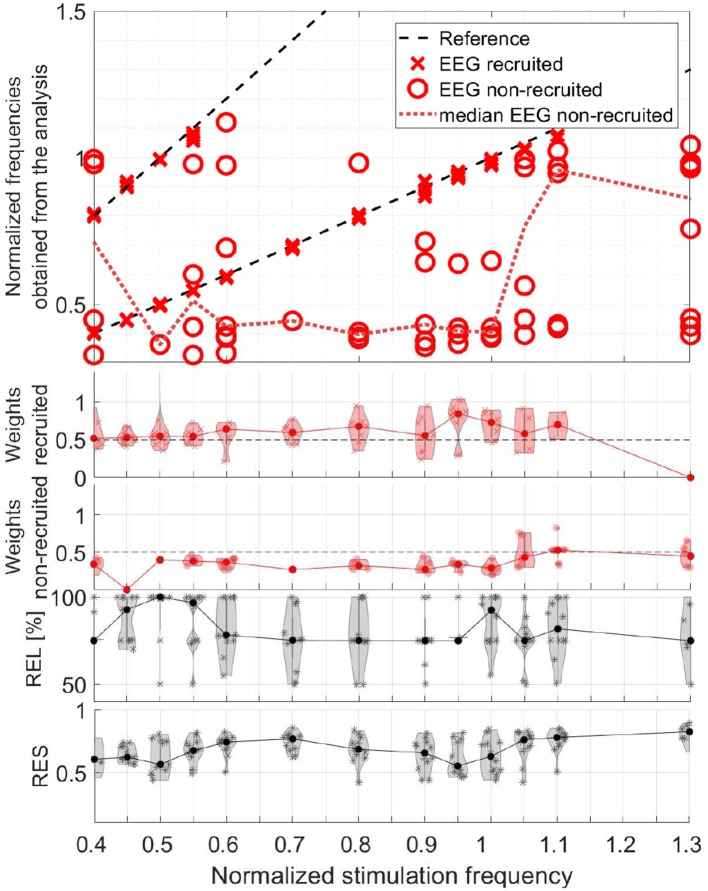
Scatter plot of the normalized frequencies obtained from the analysis for EEG data, violin plots of the weights of the recruited and non-recruited components, reliability, and residual as function of the normalized stimulation frequency.

In order to evaluate the responses of all participants together, we take the obtained frequencies, the maxima of the frequency signatures, of only the components with primarily only visual response patterns (Category 1) and differentiate the two conditions, recruited and non-recruited components. The MEG group analysis is shown in Figure 14 and the EEG group analysis in Figure 15. The second and third rows show the component weights as violin plots with the median curve, while the fourth row shows the corresponding reliability distribution and the fifth row shows the residual for each stimulation frequency.

The upper plots of both, Figures 14, 15, depict two dashed reference lines each representing the stimulation frequency *f*_s_ and its harmonic 2*f*_s_. Components very close to this line are recruited (marker X) and all others non-recruited (marker O). The results show that entrainment can appear for all of the stimulation frequencies up 1.1*f*_α_, although in each participant only a subset of stimulation experiments was successful in producing an entrainment.

The non-recruited components (dotted line denotes median) most commonly show frequencies of (0.4 ± 0.1)*f*_α_ for stimulation frequencies between 0.4*f*_α_ − 1.0*f*_α_. Between stimulation frequencies 1.0*f*_α_ − 1.3*f*_α_, response frequencies around (1.0 ± 0.05)*f*_α_ are more common. Few components appear outside this pattern with response frequencies of (0.6 − 0.7)*f*_α_. Note that the non-recruited components appear during flicker stimulation and have a clear visual response pattern. The group analysis identifies two reoccurring response frequency ranges of (0.4 ± 0.1)*f*_α_, a theta band response, and (1.0 ± 0.05)*f*_α_, an alpha band response.

It is important to note that in the components of a single measurement there are only either recruited components or non-recruited components, not both. The only rare exceptions are cases, in which the response frequency changed mid-way in the series of trials and both were superimposed during averaging. In all data sets with clear visual response pattern (Category 1) without recruitment, either the alpha or the theta band response is present, not both. Rare exceptions are again where the response frequency changes mid-way in the series of trials and both are superimposed during averaging.

The weights represent the power of each component using its norm and indicate its dominance. The violin plots of the weights in Figures 14, 15 show that the recruited components are dominant compared to the non-recruited frequencies. A comparatively high reliability and a low residual give strongest confidence in the components and their obtained frequencies for stimulation frequencies around 0.5*f*_α_ and *f*_α_. This is where the MEG and EEG signals have two components in common (REL = 100%). For the rest of the stimulation frequencies, the MEG and EEG signals have only one common component.

## 4. Discussion

### 4.1. Evaluation of C-SECSI Approach

We propose to compute the coupled CP decomposition based on the C-SECSI framework (Naskovska and Haardt, 2016). The C-SECSI framework for three-way tensors with one mode in common computes eight initial estimates, four of which are coupled and four uncoupled. The final estimate is then chosen based on the minimum reconstruction error for both tensors independently. Therefore, C-SECSI computes the coupled CP decomposition under the constraint that one of the modes is coupled, but it still computes uncoupled estimates. This is very practical for the analysis of biomedical data, where the coupling is assumed but not yet proven. Moreover, for comparing the independently chosen final estimates we have defined the coupling reliability in Equation (4). The benchmark results (Figures 3, 4) show that the reliability measure can be used to control the rank estimate of the coupled decomposition. This is a very important feature of C-SECSI, because the rank estimate is a very challenging problem, especially for noisy measurement signals. The C-SECSI framework has higher accuracy in ill-conditioned scenarios such as computing the coupled CP decomposition with a collinear factor. In both accuracy tests (Figures 5, 6), we obtained a higher accuracy than with the traditional SECSI framework proposed in Roemer and Haardt (2008, 2013) and alternative methods. Another advantage of the C-SECSI framework is that it can decompose tensors that are corrupted by noise with different variances without any additional normalization or estimation of the SNR (Figure 7).

### 4.2. Decomposition of MEG-EEG Data

The application of the coupled CP decomposition to simultaneously recorded MEG-EEG signals demonstrates its capability of extracting physiologically meaningful signal sources, in this case oscillators during photic driving. The coupled CP decomposition allows us to decompose these multidimensional heterogeneous signals into their most prominent components and residual signal. Small overestimations of the rank lead to less prominent signal patterns being extracted from the residual and represented as a component. The underlying components are extracted while preserving the original multidimensional structure of the signals (frequency × time × channels, c.f. Figure 2) under the assumption that they have a common frequency mode.

#### 4.2.1. Field-Maps

The overall rate of response in terms of a spatial activation pattern in the field-maps is comparable to preceding studies (Schwab et al., 2006; Salchow et al., 2016), which used entrainment measures to detect responses. This study, however, includes all frequencies within the wavelet frequency window around the alpha and theta band. We found a high response rate close to the individual alpha frequency, an intermediate rate in the theta band, and a low response rate otherwise. This is in agreement with Salchow et al. (2016), Schwab et al. (2006), and Halbleib et al. (2011). The EEG data present more frequent response patterns than the MEG data. This could be due to the different sensitivities to the angles and the depths of the sources in the brain (Hunold et al., 2016).

The tensor decomposition with rank 2 extracts the two primary components of the data in frequency × time × channel space and, thereby, eliminates further sources that could otherwise confound the analysis. The component topographies are either physiologically meaningful, i.e., representing a visual response, or represented some other clearly distinguishable source of similar strength. This allows us to isolate the stimulus response. In turn, this substantially improves the identifiability of visual response patterns in MEG components and to a lesser degree in EEG components. The example in Figure 12 demonstrates how a visual response component can be uncovered and isolated from an unclear MEG data set.

#### 4.2.2. Time Courses

The time signatures of the recruited components demonstrate the three phases: onset, plateau, and offset (e.g., Figure 9). This is in good agreement with previous studies (Schwab et al., 2006; Salchow et al., 2016). However, the decomposition additionally allows us to separate signal components with a weaker or instable time course (e.g., Figure 12). Further, it highlights changes over time, such as a component that is only present during the onset phase and diminishes after that (e.g., MEG in Figure 8). This component-wise time course display allows the user to gain insight into the stationarity and effectiveness of the stimulation experiment.

#### 4.2.3. Frequencies

The decomposition of the data tensor allows us to extract the main components with their frequency signatures. In this stimulation experiment, the frequency signatures primarily contain one or sometimes two peaks, which reflect the resonance to the stimulation frequency and/or intrinsic frequencies within the alpha and theta bands. Note that in general, each frequency signature can contain multiple frequency contributions, if they are related to each other in the data tensor. MEG and EEG signals do not always have all frequency components in common, this can be explained by the different sensitivities to the angles and the depths of the sources in the brain (Hunold et al., 2016) and has been observed in preceding tensor decomposition studies (Naskovska et al., 2017a,b).

The decomposition is able to separate sources at different frequencies that show the same or a similar topographical distribution in the field-map, even if the frequency peaks partly overlap (e.g., Figure 8). The existence of such similar topographies, indicating a similar physical distribution of the activity in the brain, during the plateau phase for different frequencies has been reported and described in more detail by Halbleib et al. (2011). The strongest resonance frequency peak during stimulation is in many cases slightly above the alpha frequency peak at rest, i.e., 1*f*_α_ − 1.1*f*_α_. This can be understood from the observation that the signal contribution to the alpha band above the peak frequency, the upper alpha, and the signal contribution below the peak, the lower alpha, respond independently (Klimesch, 1999). Our results indicate that the strongest resonance in this case may be produced by stimulation in the upper alpha range. A desynchronization of the lower alpha band is associated with attention (Klimesch, 1999). The attention of the participant may, therefore, play a role in the composition of the power distribution in the alpha band as well. In the example in Figure 8, Component 2 (red) reflects the resonance frequency in the upper alpha range, while Component 1 (blue) reflects the remaining signal contributions to the alpha band, primarily in the lower alpha range. The separation is more pronounced in the MEG data than the EEG data in this case.

During stimulation with a frequency of approximately *f*_*s*_ = 0.5*f*_α_, we can differentiate two components, one with a frequency peak at the stimulation frequency *f*_*s*_ and one with its harmonic 2*f*_*s*_ ≈ *f*_α_, e.g., in Figure 9. This is in agreement with Salchow et al. (2016), who determined alpha entrainment from the 2*f*_*s*_ ≈ *f*_α_ peak amplitude. The critical feature of this case is that the frequency peaks match closely the stimulation frequency and its harmonic (dashed lines in Figures 14, 15).

### 4.3. Oscillators During Photic Stimulation

#### 4.3.1. Recruitment

The unsupervised tensor decomposition of MEG-EEG signals isolates components that are characterized by one frequency peak, respectively, which is sustained through all or part of the stimulation train. These can be understood as oscillations. Within the components displaying clear visual response topographies (Category 1), approximately one third of the MEG components and approximately half of the EEG components are recruited to the stimulation frequency or its first harmonic (Figures 14, 15). This concurs with the entrainment reported in previous studies (Mangan et al., 1993; Klimesch, 1999; Herrmann, 2001; Schwab et al., 2006; Salchow et al., 2016). Using photopic stimulation conditions, Herrmann (2001) additionally describes entrainment for higher harmonics. The observation that in a single experiment we can only observe either a recruited oscillation or a non-recruited oscillation, but not both, suggests that the oscillators underlying the non-recruited oscillations are entrained to the stimulation frequency in the recruited case. This is supported by Wacker et al. (2011) and Halbleib et al. (2011), who point out that these recruited oscillations are not purely synchronized stimulus responses, but reflect a driven oscillator in the brain that maintains the oscillation for several cycles after the stimulation has ended. Notbohm et al. (2016) further strengthen this hypothesis using jittered flash stimulation experiments.

#### 4.3.2. Intrinsic Oscillations

The successful visual stimulation also identifies components with non-recruited frequencies, which are approximately two thirds of the MEG components and approximately half of the EEG components. These can be considered intrinsic oscillations (da Silva, 1991), because they are produced without a match in stimulation frequency and occur across participants. They appear primarily in two frequency bands, 0.9*f*_α_ − 1.1*f*_α_, the alpha band, and 0.3*f*_α_ − 0.5*f*_α_, the theta band (Figures 14, 15). Although MEG and EEG have partly different sensitivities, the group analysis across participants of both modalities confirm this finding. The presence of alpha band responses during stimulation with other frequencies is confirmed by Herrmann (2001). Theta band oscillations during photic stimulation have been described in children (Lazarev et al., 2001) and in students (19–24 years; Mangan et al., 1993) similar to our participants.

#### 4.3.3. Resonance

The response amplitude in the tensor components resonates for stimulation frequencies in the alpha band and the theta band. Resonance is also observed for stimulation close to half of the individual alpha frequency, which may partly coincide with the theta band response. The resonance to stimulation in the alpha band and half of it is in good agreement with existing literature (Herrmann, 2001; Schwab et al., 2006; Salchow et al., 2016). The resonance in the theta band is confirmed and described by Mangan et al. (1993), Lazarev et al. (2001), and Klimesch (1999). In agreement with these studies, our results show that the resonance extends to stimulation frequencies that are a small fraction above and below the respective intrinsic frequency peak.

#### 4.3.4. Connections

The observation that in the non-recruited case we observe either a component in the alpha band or a component in the theta band, not both, indicates a reciprocal relation between the two bands. In fact, such a reciprocal relation in band power has been reported in the EEG literature and summarized in a comprehensive review by Klimesch (1999). Our study confirms this observation in MEG signals. da Silva (1991) hypothesizes that the brain contains a system of connected neural oscillators with individual resonance frequencies that respond to photic driving. Our results support this hypothesis and suggest alpha and theta oscillators that can resonate with photic driving. When an entrainment to a stimulation frequency takes place, then the recruited frequencies dominate the signals.

### 4.4. Future Work and Applications

The time-frequency-channel tensor in this study translates well to other studies in neuroscience in that it uses typical data dimensions of time, e.g., a measurement, channels, e.g., a set or array of sensors, and a series of features or parameters, e.g., frequencies. The decomposition method can be applied to the unsupervised discovery of patterns in data of exploratory studies in the future. The component analysis can be extended by performing source reconstructions of the components. This could reveal the locations of the underlying brain activity regions. It could also allow for quantitative measures of the physiological integrity of the components. This may help in providing new insight into the brain's organization and function. Future work could involve higher-order tensors, for example, by including the participant as one dimension of a population tensor. The necessary cross-participant co-registration and normalization should be investigated and validated.

The tensor decomposition approach can be of high practical value when integrated into brain-computer-interfaces, such as a speller application for paralyzed users (Rezeika et al., 2018). Such spellers detect user selections from the brain's response to steady state visual stimulation. Using the selections, the user can navigate through a list of letters on a screen and produce a message. Another practical use is in neurofeedback systems, which decompose and display features of the measured brain activity on-line to the user and allow the user to train control over beneficial brain states. In the clinical setting, it can augment and further contribute to diagnostics. It can lead to improved treatment outcome of brain disorders, for example, by locating epileptic network nodes in the epileptic brain.

Beyond neuroscience, the tensor decomposition method is highly applicable to machine learning tasks in big data in general, specifically the discovery of multi-dimensional patterns (Stoudenmire, 2018; Klus and Gelβ, 2019).

## 5. Conclusion

The tensor decomposition with C-SECSI is able to separate physiologically meaningful oscillations of visually evoked brain activity from background signals. The component frequencies identify either an entrainment to the respective visual stimulation frequency or its first harmonic, or an oscillation in the individual alpha band or theta band. In the group analysis of both, MEG and EEG data, a reciprocal relationship between alpha and theta band oscillations is present. The coupled tensor decomposition using the C-SECSI framework is a robust, powerful method for the unsupervised extraction and separation of meaningful sources from multidimensional biomedical measurement data.

## Data Availability Statement

The datasets generated for this study are available on request to the corresponding author.

## Ethics Statement

The studies involving human participants were reviewed and approved by Faculty of Medicine of the Friedrich-Schiller-University Jena, Germany. The patients/participants provided their written informed consent to participate in this study.

## Author Contributions

All authors designed the research. KN, AK, and MH developed the decomposition method. KN, SL, and AK performed the data analysis. All authors contributed to the scientific discussion. SL and KN compiled the paper with contributions from all other authors.

### Conflict of Interest

The authors declare that the research was conducted in the absence of any commercial or financial relationships that could be construed as a potential conflict of interest.
